# Arbuscular Mycorrhizal Fungi: Boosting Crop Resilience to Environmental Stresses

**DOI:** 10.3390/microorganisms12122448

**Published:** 2024-11-28

**Authors:** Wenjing Nie, Qinghai He, Hongen Guo, Wenjun Zhang, Lan Ma, Junlin Li, Dan Wen

**Affiliations:** 1 Yantai Key Laboratory of Evaluation and Utilization of Silkworm Functional Substances, Yantai Engineering Research Center of Plant Stem Cell Targeted Breeding, Shandong Engineering Research Center of Functional Crop Germplasm Innovation and Cultivation Utilization, Shandong Institute of Sericulture, Yantai 264001, China; 2 State Key Laboratory of Nutrient Use and Management, Shandong Key Laboratory of Bulk Open-Field Vegetable Breeding, Ministry of Agriculture and Rural Affairs Key Laboratory of Huang Huai Protected Horticulture Engineering, Institute of Vegetables, Shandong Academy of Agricultural Sciences, Jinan 250100, China; 3 Shandong Fruit Research Institute, Tai’an 271000, China

**Keywords:** arbuscular mycorrhizal fungi (AM fungi), abiotic stress, biotic stress, plant resistance, ecosystem health

## Abstract

Amid escalating challenges from global climate change and increasing environmental degradation, agricultural systems worldwide face a multitude of abiotic stresses, including drought, salinity, elevated temperatures, heavy metal pollution, and flooding. These factors critically impair crop productivity and yield. Simultaneously, biotic pressures such as pathogen invasions intensify the vulnerability of agricultural outputs. At the heart of mitigating these challenges, Arbuscular Mycorrhizal Fungi (AM fungi) form a crucial symbiotic relationship with most terrestrial plants, significantly enhancing their stress resilience. AM fungi improve nutrient uptake, particularly that of nitrogen and phosphorus, through their extensive mycelial networks. Additionally, they enhance soil structure, increase water use efficiency, and strengthen antioxidant defense mechanisms, particularly in environments stressed by drought, salinity, extreme temperatures, heavy metal contamination, and flooding. Beyond mitigating abiotic stress, AM fungi bolster plant defenses against pathogens and pests by competing for colonization sites and enhancing plant immune responses. They also facilitate plant adaptation to extreme environmental conditions by altering root morphology, modulating gene expression, and promoting the accumulation of osmotic adjustment compounds. This review discusses the role of AM fungi in enhancing plant growth and performance under environmental stress.

## 1. Introduction

Against the backdrop of global climate change and environmental degradation, agricultural production confronts a myriad of challenges. Extreme weather conditions such as high temperatures, salinization, drought, and environmental pollution profoundly impact soil health and crop vitality. Elevated temperatures can induce heat stress in plants, disrupting their physiological metabolism and photosynthesis, consequently diminishing growth rates and yields [1]. Drought severely limits crop growth by restricting the effective supply of water and nutrients [2]. Salinization impairs root functionality and disrupts intracellular ion balances [3], while heavy metal contamination hinders photosynthesis, growth, and development [4]. Concurrently, environmental stresses increase susceptibility to pests and diseases, weakening plant defense mechanism and exacerbating the incidence and severity of these afflictions [5,6].

**Figure 1:**
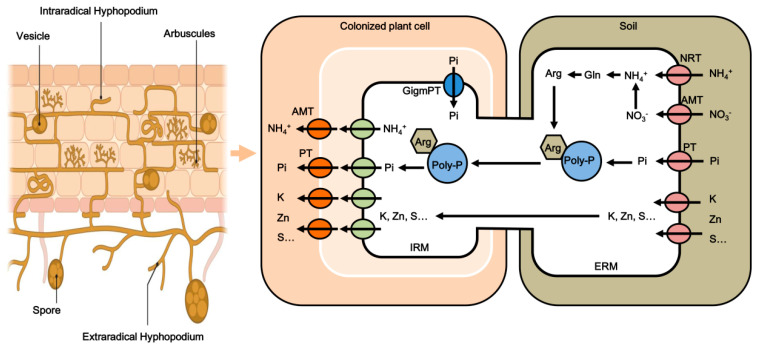
Schematic diagram of AM fungi structure and nutrient uptake pathways. This diagram illustrates the anatomical structure of AM fungi within the root cortex of host plants and highlights the nutrient uptake pathways involved in symbiosis. The extraradical mycelium (ERM) of AM fungi plays a crucial role in the uptake of phosphate (Pi), utilizing specialized fungal phosphate importers from the surrounding soil. Additionally, ammonium (NH_4_^+^) and nitrate (NO_3_^−^) are absorbed by the ERM and assimilated into glutamine (Gln) and subsequently converted to arginine (Arg^+^). This assimilation process results in the production of either excess H^+^ with ammonium or OH^−^ with nitrate. Phosphate is primarily transported in the form of polyphosphate granules, the negative charge of which aids in the simultaneous transport of arginine and various metal ions from the ERM to the intraradical mycelium. The efflux mechanisms of Pi and NH_4_^+^ from the intraradical mycelium (IRM) to the ERM are not well understood and require further research, as does the uptake of metal ions by the ERM, which necessitates additional study into the transport proteins of both the host plant and the AM fungi. (The schematic diagram of AM fungi structure in this figure was created using BioRender).

Arbuscular mycorrhizae (AM fungi), a widespread and ancient symbiosis between soil fungi and plant roots, are prevalent across various terrestrial plants including angiosperms, gymnosperms, and ferns [7]. These symbiotic associations are particularly notable for their role in plant evolution and adaptation, with AM fungi’s origins dating back to the Early Devonian period, roughly 400 to 450 million year ago [8]. This period is crucial as it marks the transition of plants from aquatic to terrestrial environments [9,10,11]. AM fungi are obligate symbionts that depend entirely on their host plants for survival and reproduction, unable to sustain independently. While host plants are not strictly dependent on AM fungi, these AM fungi are essential for their own reproductive and developmental processes. AM fungi are predominantly located within the root cortex cells of a vast majority of plant species. Structurally, as shown in Figure 1, AM fungi are characterized by arbuscules, which form within the plant roots post-infection and are essential for the symbiotic relationship. Additionally, AM fungi develop vesicles—spherical or elliptical, thin-walled structures filled with lipids, serving both as storage and potentially as reproductive structures [12]. The network of AM fungi extends beyond the root with extraradical and intraradical hyphae, creating a vital link between the plant and soil, which is integral for nutrient exchange [12].

The critical role of AM fungi in symbiosis was notably advanced by Akiyama et al. [13] in 2005, who identified the plant hormone strigolactone (SL) as a key inducer of hyphal branching in AM fungi, representing a significant breakthrough in understanding their symbiotic mechanisms. In this mutually beneficial relationship, plants provide the essential carbon sources required by AM fungi, while in return, AM fungi significantly enhance the plants’ nutrient absorption capabilities from the soil, particularly vital in conditions of nutrient scarcity [14,15]. Studies have demonstrated that AM fungi effectively enhance the uptake of phosphorus (P) and nitrogen (N), especially in nutrient-limited environments [16,17,18]. AM fungi enzymatically break down organic P in the soil, channeling inorganic P to the host plant through their mycelial network, which is particularly critical when soil P concentrations are low. This activity substantially increases P uptake and improves crop yields [19]. As shown in Figure 1, P absorption in plants primarily occurs via two pathways: directly through the AM fungi mycelium and indirectly through P transport proteins on the root hairs and epidermal cell membranes [20]. Additionally, AM fungi aid in the absorption of N, predominantly in the forms of NH_4_^+^ and NO_3_^−^, including amino acids, with NH_4_^+^ being the primary form [21,22]. They also facilitate the uptake of other essential nutrients such as sulfur (S), copper (Cu), and zinc (Zn) [23]. AM fungi exert a profound influence on plant biomass, growth, and resilience against environmental stresses and pathogens, thereby playing a critical role in maintaining ecosystem balance and stability. Comprehensive research has established that AM fungi are essential for plant adaptation to abiotic stresses such as drought, low temperatures, and saline–alkali environments. By enhancing nutrient uptake, AM fungi significantly improve plant tolerance to drought, salinity, and heavy metal stress by optimizing water use efficiency and modulating physiological metabolic processes [8,12,24,25,26,27,28,29,30]. Moreover, AM fungi activate the plant immune system, thereby increasing resistance to soil-borne pathogens and nematodes and enhancing crop safety and quality [19,20,21,22,31]. They also protect root systems from various soil-borne pathogens, acting as biological control agents through a combination of physical, chemical, and biological mechanisms [23,32,33,34].

As symbionts, AM fungi are vital for ecosystem restoration and improving plant adaptability. Widely distributed across terrestrial ecosystems, AM fungi display biogeographical patterns that reflect the influences of latitude, climate, evolutionary relationships, and biological dispersal. Within the soil, the mycelial networks of AM fungi enhance soil structure and facilitate the biogeochemical cycling of crucial elements such as nitrogen, carbon, and phosphorus, which are indispensable for sustaining ecosystem functions and promoting sustainable agricultural practices [7,21,22,23,24,25,26,35,36].

While research on AM fungi has highlighted their considerable potential for modern agriculture, numerous technical and economic challenges persist in their practical application. The diversity of AM fungi species and their specific interactions with certain host plants necessitate further investigation [37,38,39]. In addition, methods for selecting and propagating AM fungi strains need refinement to boost their stability and effectiveness across diverse agricultural ecosystems [40,41,42,43,44]. This article delves into the functions and mechanisms of AM fungi, examines the main challenges faced in agricultural production, and discusses prospective research and application directions. The aim is to provide a comprehensive resource for agricultural scientists and technicians involved in both theoretical research and practical application, facilitating a deeper understanding and effective utilization of AM fungi in agriculture.

## 2. Effects of AM Fungi on Plant Drought Tolerance

Drought stress typically results in a diminished soil water potential, leading to the dehydration of plant cells, constraints on cell expansion and division, diminished leaf area, restricted stem elongation, inhibited root development, and impaired stomatal functions. Collectively, these effects result in a reduced efficiency of water and nutrient absorption [45].

Infection by AM fungi enhances the morphology of the host plant’s root system, evidenced by an increased total root length, number of lateral roots, and root surface area, which in turn improve the plant’s water and nutrient uptake capabilities. Studies by Liu et al. (2016) [46] and Comas et al. (2013) [47] have demonstrated that trifoliate orange seedlings infected with AM fungi display enhanced root system morphology under drought conditions, facilitating a more efficient use of soil resources. Moreover, AM fungi can modulate plant hormonal responses to drought, adjusting levels of hormones such as abscisic acid (ABA) and ergosterol [48]. For instance, in citrus sinensis seedlings, inoculation with *Funneliformis mosseae* AM fungi significantly elevates the levels of indoleacetic acid (IAA), ABA, methyl jasmonate (MeJA), and zeatin riboside (ZR) under drought stress, thereby augmenting the plant’s drought resilience [46]. Furthermore, AM fungi facilitate the plant’s osmotic adjustment mechanisms by promoting the accumulation of both inorganic ions and organic solutes, such as glucose, fructose, and sucrose, which assist in maintaining cellular osmotic balance under drought conditions [49]. This accumulation not only alleviates stress from cellular dehydration but also protects cellular structures and functions.

AM fungi enhance plants’ ability to maintain osmotic balance and mitigate stress damage by boosting the accumulation of osmotic regulatory substances, including proline, glucose, and sucrose. Research indicates that plants colonized by AM fungi exhibit significantly higher levels of these osmotic regulatory substances compared to non-AM fungi plants under drought conditions [50,51,52]. AM fungi significantly enhance crop resilience to drought stress through a series of physiological and molecular adaptations. These adaptations include improved photosynthetic efficiency and osmotic adjustment, leading to enhanced survival in arid conditions. AM fungi alleviate damage to photosynthetic organs caused by drought by diminishing the accumulation of reactive oxygen species (ROS) and bolstering antioxidant defense mechanisms. Remarkably, AM fungi-inoculated *Rosa damascena* and *Citrullus lanatus* exhibit increased chlorophyll content and superior photosynthetic efficiency under drought conditions, as evidenced by various studies [53,54]. Furthermore, AM fungi optimize carbon dioxide uptake and utilization by increasing stomatal conductance and enhancing the efficiency of photosystem II (PSII) [55]. Additionally, they enhance plant resistance to oxidative stress by upregulating genes associated with antioxidant defenses, including superoxide dismutase (SOD), catalase (CAT), and peroxidase (POD) [56,57]. This augmented antioxidant response effectively reduces drought-induced cellular damage while promoting normal plant growth and development. Moreover, AM fungi bolster the antioxidant capacity of plants by activating key antioxidant enzymes such as SOD and CAT, thereby decreasing ROS accumulation under conditions of drought stress [58]. Research by Zou et al. (2015a) further reveals that AM fungi can regulate water and hydrogen peroxide transport in the root system by modulating the function of aquaporins [59].

AM fungi significantly enhance water uptake in host plants by expanding and branching their extraradical mycelium, which taps into deeper soil layers and alleviates water scarcity [60,61]. The mycelial networks of AM fungi also play a pivotal role in stabilizing soil aggregates, essential for the formation and maintenance of large, water-stable soil aggregates crucial for carbon sequestration [62,63]. Additionally, AM fungi modify the architecture of plant root systems, increasing their surface area and improving water and nutrient absorption, particularly under drought conditions. Studies have demonstrated that mycorrhizal seedlings develop elongated root hairs, significantly enhancing their drought tolerance [64]. Aroca et al. (2007) evaluated the influence of AM symbiosis on the hydraulic properties, aquaporin expression, and root proliferation in soybean roots under various stress conditions, including drought, cold, and salinity. Their findings indicate that colonization by *Glomus intraradices* AM fungi prevents leaf dehydration during drought and salt stress by maintaining a higher relative water content in AM plants compared to their non-AM counterparts [65].

At the molecular level, AM fungi modulate the gene expressions involved in water and nutrient transport. They promote the expression of aquaporin genes, pivotal for regulating transmembrane water movement, thereby enhancing cellular water management and adaptability to drought conditions [66,67]. Following inoculation with AM fungi, significant upregulation of aquaporin expression has been observed in citrus and tomato, directly correlating with improved water uptake capacity and enhanced drought resilience. Furthermore, AM fungi influence the expression of water channel proteins across a variety of plant species, including *Phaseolus vulgaris*, *trifoliate orange*, and olive (*Olea europaea* L.) [16,65,68]. AM fungi also modify the expression of genes encoding water channel proteins in plant root systems, such as plasma membrane intrinsic proteins (PIPs) and members of the aquaporin gene family, thus improving the plants’ water uptake and cellular water regulation under drought conditions [69,70].

Beyond enhancing water uptake, mycorrhizal symbiosis also indirectly improves nutrient acquisition under drought conditions [71]. In dry soils, the exploratory capacity of plant roots is constrained, limiting nutrient absorption. Mycorrhizal symbiosis bolsters the host plant’s capacity to absorb and transport mineral nutrients, especially in nutrient-deficient soils [72]. This enhancement is facilitated by the finer diameter of mycelium compared to the plant root system, which enables it to circumvent zones of nutrient depletion and penetrate small soil voids to access essential nutrients [73]. Additionally, the secretion of phosphatases into the soil is increased, either directly through the extraradical mycelium [74] or by inducing the exudation of root phosphatases [75]. This action promotes the decomposition of organic orthophosphates, alleviating the impacts of drought stress on plant nutrient dynamics. Studies have demonstrated that AM fungi improve plant growth under drought by enhancing the uptake of critical elements such as nitrogen (N), phosphorus (P), potassium (K), and magnesium (Mg). Inoculation with Glomus versiforme and G. mosseae significantly increased the N, P, and K content in rabbit-eye blueberries (*Vaccinium ashei Aiton*.), promoting their growth [76]. Mixed inoculation with AM fungi also enhanced the uptake of P, K, Na, and Ca in carob (*Ceratonia siliqua* L.) [77]. Inoculations with Rhizophagus irregularis and *Funneliformis mosseae* improved nutrient uptake in maize and tomato (*Solanum lycopersicum* L.) [78], and inoculation with the heteromorphic root ascomycete increased the macro- and micronutrient concentration in Populus euphratica 107 and the P content in Ningxia wolfberry (*Lycium barbarum* L.), supporting plant growth under drought stress [79,80]. Under drought conditions, AM fungi significantly enhance the nutrient uptake capabilities of plants, particularly for nitrogen, phosphorus, potassium, and calcium. Wu et al. reported that plants associated with AM fungi exhibit superior nutrient uptake and water transport capacities under drought stress compared to non-mycorrhizal plants [81,82].

**Figure 2:**
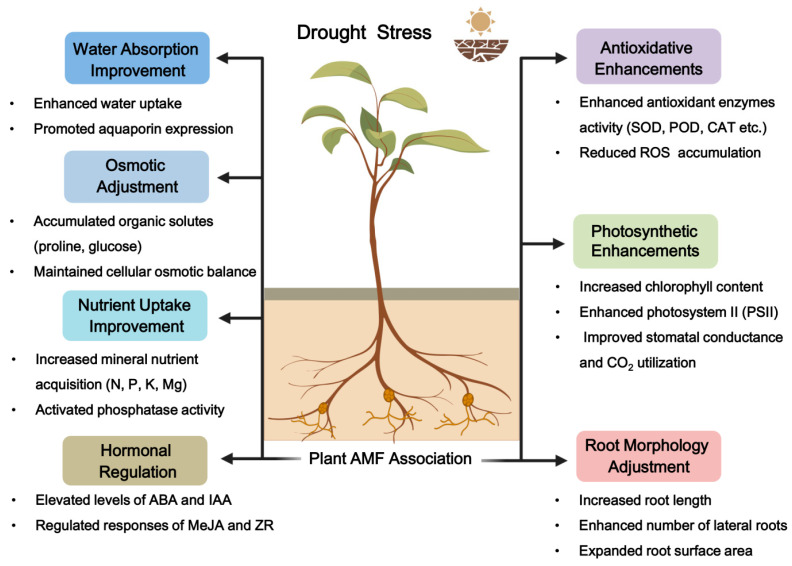
Mechanisms of action of AM fungi in enhancing plant resistance to drought stresses. (All elements, except for the text, were created using BioRender.).

Table 1 summarizes the impact of AM fungal inoculation on the drought resilience of various plant species. Figure 2 elucidates the principal mechanisms through which AM fungal inoculation enhances drought resistance in plants. By leveraging these diverse mechanisms, AM fungi significantly enhance plant survival and adaptability in arid conditions, presenting a promising biological strategy to support sustainable agricultural practices in drought-affected regions. 

## 3. Effects of AM Fungi on Plant Salt Tolerance

Soil salinization represents a significant global trend in soil degradation, severely impacting crop growth, particularly in arid and semi-arid regions. As crucial components of natural ecosystems, arbuscular mycorrhizal (AM) fungi form beneficial symbiotic relationships with plants and thrive in saline environments, enhancing plant productivity. In soils impacted by salinity and sodicity, where poor drainage often leads to surface salt accumulation, plant growth is adversely affected. AM fungi are widely present in these environments, and research has shown that salinity influences not only the formation and function of the mycorrhizal symbiosis but also indicates that plants colonized by AM fungi in saline soils exhibit significantly higher productivity than non-colonized plants [91,92,93,94,95].

Under salt stress conditions, high salt concentrations lead to significant Na^+^ accumulation within plant cells, impeding water absorption and causing osmotic potential imbalances both within and outside the cell, potentially leading to cell wall separation. To counteract these effects, plants synthesize osmoregulatory substances such as proline, soluble sugars, betaine, and polyamines, which help mitigate the osmotic pressure differences and prevent dehydration [96]. AM fungi support plants in resisting salt stress by promoting the synthesis of these substances. For example, it has been observed that proline accumulation in cotton grown under conditions of slightly, moderately, highly, and extremely saline soils was significantly enhanced by inoculation with AM fungi [97]. Additionally, soluble sugars like glucose and sucrose, which serve as carbon storage substances, are increased in concentration by the degradation of starch under salt stress conditions, aiding in maintaining the osmotic balance inside and outside of cells [98]. Numerous studies have documented that AM fungi can promote proline accumulation in plants. However, under the same salt conditions, some research has noted that the proline concentration in the leaves of AM fungi-inoculated plants is lower than in uninoculated plants. This reduction may be attributed to the role of AM fungi in alleviating salt stress, which includes reducing imbalances in osmotic potential across the cell membrane and decreasing the production of reactive oxygen species (ROS) in plants [99]. Trehalose, a non-reducing storage disaccharide that regulates carbohydrate metabolism, can also alleviate the effects of salt stress through AM fungal intervention. AM fungi have been shown to enhance the activity of 6-phospho-trehalose synthase in pigeon peas, increasing the trehalose content in the aerial parts of the plant [100]. Furthermore, osmoregulatory substances such as polyamines and organic acids also play crucial roles in regulating intracellular ion balance and enhancing plant resilience to adverse conditions.

Salinized Soils and nutrient dynamics-salinized soils are characterized by high concentrations of sodium (Na^+^) and chloride (Cl^−^), which disrupt the absorption and transport of essential nutrients such as potassium (K^+^) and magnesium (Mg^2+^), leading to nutrient deficiencies and ionic imbalances in plants [96]. Arbuscular mycorrhizal (AM) fungi play a pivotal role in enhancing plant nutrient absorption, notably phosphorus (P), nitrogen (N), zinc (Zn), copper (Cu), and iron (Fe), thereby increasing plant resilience against salt stress [101,102,103,104,105,106,107]. These fungi improve nutrient uptake primarily through modifications to root architecture, which can increase root surface area and length, facilitating greater nutrient absorption [108]. Wu et al. found that citrus trees inoculated with AM fungi exhibited significantly enhanced root development compared to their uninoculated counterparts [109]. Moreover, AM fungi can absorb substantial amounts of a plant’s total P and N—up to 80% and 25%, respectively—through their extensive mycelial networks [110]. The enhancement of P nutrition is crucial for assisting plants in coping with saline stress, although AM fungi also support other physiological processes that promote growth under such conditions [92,93,111,112,113]. In saline soils, the contribution of P from AM fungi is particularly significant as P tends to bind with soil cations like calcium, leading to precipitation and reduced bioavailability, thus impeding plant growth [108]. These fungi also enhance P acquisition by secreting enzymes such as phosphatases, which release P into the soil, and by possessing proteins that allow for efficient P uptake even at low concentrations [108].

It has been reported that high soil Na^+^ levels inhibit plant growth primarily by causing K^+^ to be replaced with Na^+^ in plant tissues, which disrupts numerous physiological processes [114,115,116]. Increased Na^+^ in the soil solution interferes with various transport proteins in the root plasma membrane, such as K^+^-selective ion channels, reducing the uptake of K^+^ and other essential nutrients. This also restricts root development and overall plant growth [117]. Consequently, the uptake of water and minerals like P, K, Fe, Cu, and Zn is diminished, as is the population of soil bacteria [118]. The ratio of potassium to sodium (K^+^/Na^+^) is a critical metric for assessing the salt tolerance of plants, including tomato cultivars [119]. Additionally, a high Na^+^/K^+^ ratio can disrupt key metabolic processes, such as protein synthesis in the cytoplasm [120]. Giri et al. (2007) observed that the concentration of K^+^ in the tissues of gum arabic plants colonized by *G. fasciculatum* was higher at all salinity levels tested, indicating that AM fungi can help maintain a favorable K^+^/Na^+^ balance, which is essential for preventing disruptions in enzymatic reactions and protein synthesis [95]. These fungi also influence the mineral nutrient content of plants under salt stress by promoting and selectively absorbing nutrients, which helps plants to maintain critical ion balances and enhance overall mineral nutrition [121,122,123,124].

AM fungi facilitate plant adaptation to saline–alkali conditions by modulating specific ion transport proteins within plant cells, such as the Na^+^/H^+^ antiporter (NHX). This transporter is crucial for moving Na^+^ from the cytoplasm into vacuoles, thereby reducing cytoplasmic Na^+^ concentrations and maintaining intracellular ionic balance and functionality [125]. Under salt stress, rice plants symbiotic with AM fungi show an enhanced vacuolar sequestration of Na^+^ by upregulating the *OsNHX3* gene expression in their leaves. Simultaneously, the expression of the *OsSOS1* gene is also enhanced, which helps transfer Na^+^ from the cytoplasm to the extracellular space, effectively maintaining low intracellular Na^+^ levels and improving salt tolerance [125].

AM fungi enhance the efficiency of CO_2_ exchange with the atmosphere, contribute to transpiration, and play a significant role in regulating stomatal conductance in leaves. They improve ionic balances, protect enzymatic activities, promote water absorption, and facilitate the regulation of osmotic balance and carbohydrate composition in plants [95,126,127].

Plants in saline soils often experience physiological drought due to high salt concentrations in the rhizosphere, which reduce soil water potential and hinder water absorption [108]. However, plants symbiotic with AM fungi typically exhibit higher relative water contents. Chen et al. reported that under salt stress conditions, plants inoculated with AM fungi maintained a higher relative water content, likely due to morphological changes in the plants’ root systems and the strong water-absorbing capability of the fungal hyphae [128]. Furthermore, AM fungi regulate the expression of aquaporin genes in plant root cells, enhancing water uptake and contributing to improved drought resilience.

AM fungi can significantly improve the antioxidant capacity of plants and mitigate oxidative damage caused by salt stress. Research has demonstrated that plants inoculated with AM fungi show enhanced growth and increased activity of antioxidant enzymes, as well as reductions in relative electrolyte conductivity (REC), malondialdehyde (MDA) concentration, and the accumulation of reactive oxygen species (ROS) in leaves [129]. Additionally, under NaCl stress, inoculation with AM fungi activates antioxidant enzyme systems such as superoxide dismutase (SOD) and peroxidase (POD) in rice, enhances osmotic adjustment capacity, and reduces MDA content [130]. Another study indicated that coating wheat (*Triticum aestivum*) seeds with AM fungi prior to sowing, and subsequent cultivation under salt stress conditions, increases the activity of antioxidant enzymes including ascorbate peroxidase (APX), SOD, POD, and catalase (CAT) in seedlings. This treatment also leads to decreased accumulation of hydrogen peroxide (H_2_O_2_), reduced electrolyte permeability, and lower MDA content in the seedlings [131].

**Figure 3:**
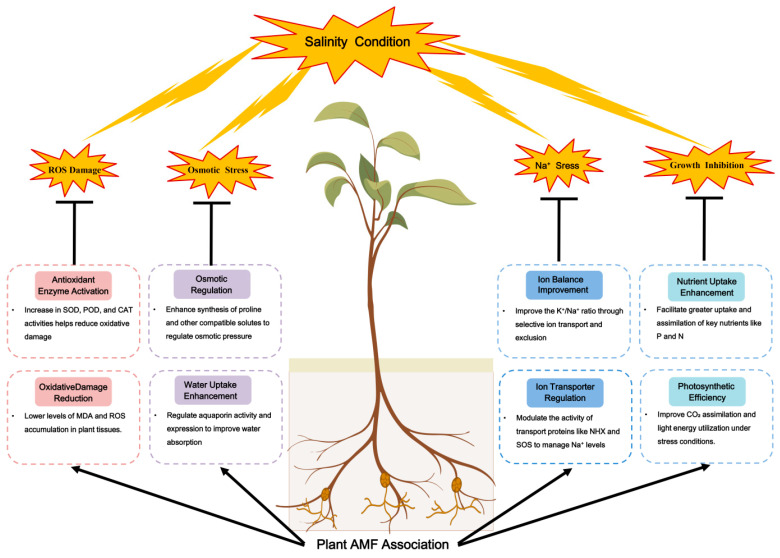
Mechanisms of action of AM fungi in enhancing plant resistance to salt stresses. (The representations of the plant, mycorrhizae, and soil were created using BioRender).

AM fungi substantially improve the productivity of saline-alkali soils and play a pivotal role in enhancing nutrient uptake by plants in these challenging environments. Table 2 details the impact of AM fungi on the salt tolerance of diverse crops, highlighting their active participation in a spectrum of physiological and ecological processes that categorize them as integral biological modifiers of saline-alkali soils. As illustrated in Figure 3, AM fungi mitigate the adverse effects of salt stress on plant physiology by modulating rhizosphere ion dynamics and nutrient absorption, reducing Na^+^ toxicity, preserving osmotic balance, promoting photosynthesis, reinforcing antioxidant systems, augmenting water uptake, and orchestrating the molecular mechanisms of gene and protein regulation [108,132].

## 4. Effects of AM Fungi on Temperature Stress

Temperature profoundly influences plant growth, development, and geographical distribution. Plants dynamically adjust their growth and developmental processes in response to fluctuations in external environmental temperatures. Cold stress, recognized as a critical abiotic factor, significantly impairs the growth and productivity of a wide range of crops worldwide. This stress is characterized by decreased metabolic rates, impaired cell membrane function, solute leakage, protein degradation, accelerated sugar metabolism, and reproductive failures. The use of AM fungi to mitigate cold stress is increasingly considered a viable strategy due to its proven effectiveness in enhancing plant performance under both normal and stress conditions. Notably, even though root colonization by AM fungi is diminished at temperatures below 15 °C, symbiotic associations with AM fungi have demonstrated a capacity to increase cold tolerance in plants. This symbiotic interaction contributes to reduced lipid peroxidation, preserved cell membrane integrity, elevated antioxidant activity, optimized osmolyte accumulation, enhanced root hydraulic regulation, increased rates of photosynthesis and respiration, and improved overall cold tolerance through the coordinated regulation of genes responsive to cold stress [148,149,150].

Research has shown that AM fungi significantly bolster cold tolerance in various crops via diverse physiological and biochemical pathways. Devi et al. (2019) found that AM fungi ameliorate cold stress in plants by reducing lipid peroxidation, maintaining membrane integrity, and augmenting antioxidant capacity, thereby facilitating superior osmotic adjustment and enhancing root hydraulic conductivity. This ultimately elevates plant performance under conditions of cold stress [150]. Furthermore, Liu et al. (2023) observed that a consortium of AM fungi strains improves growth and photosynthetic efficiency in peanuts, in addition to boosting the activity of antioxidant enzymes, which play a crucial role in managing oxidative stress induced by cold [129]. Similarly, Ma et al. (2015) and Ma et al. (2019) demonstrated that AM fungi aid in the uptake of nutrients, particularly phosphorus, thus supporting enhanced growth and stress response in cucumber seedlings, even at suboptimal temperatures [151,152].

Studies such as those by Pasbani et al. (2020) [153] showed that AM fungi inoculation in eggplants activated antioxidant defense systems and promoted the accumulation of protective molecules, thus mitigating the impacts of cold stress. Chu et al. (2016) [154] found that in Elymus nutans, AM fungi inoculation reduced oxidative damage under cold stress through increased activities of key antioxidant enzymes like superoxide dismutase and catalase. Collectively, these findings underscore the significant role of AM fungi in bolstering crop tolerance to low temperatures by enhancing photosynthetic activity, strengthening antioxidant defenses, and improving nutrient and water uptake efficiency, leading to improved growth and sustainability in adverse conditions.

AM fungi also play a pivotal role in enhancing crop resilience to heat stress, an issue of growing importance due to global climate change. Reva et al. [155] demonstrated that AM fungi inoculation improved the endurance, productivity, and fruit quality of tomatoes, peppers, and cucumbers under heat stress by fostering sustainable agricultural practices. Cabral et al. (2016) [156] reported that AM fungi altered nutrient allocation in wheat, enhancing grain yield under heat conditions by modulating nutrient dynamics. In cyclamen, Maya and Matsubara [157] noted that colonization by Glomus fasciculatum significantly increased biomass production and reduced leaf browning by enhancing the plant’s antioxidative capacity under heat stress. Additionally, studies by Wei et al. (2023) [158] on perennial ryegrass demonstrated that the combined application of AM fungi and melatonin markedly improved plant physiological responses to heat, such as increased photosynthesis and reduced leaf senescence. Yeasmin et al. (2019) [159] also highlighted that AM fungi inoculation in asparagus not only boosted growth and nutrient uptake but also activated antioxidative enzymes, thereby mitigating heat-induced oxidative damage. Moreover, Duc et al. (2018) [160] explored the synergistic effects of AM fungi in tomatoes under combined drought and heat stress, where specific AM fungi notably enhanced stomatal conductance and photosynthetic efficiency, reducing oxidative stress. Table 3 enumerates the impacts of inoculating various plant species with AM fungi on their resilience to both high and low temperature extremes. Collectively, these investigations reveal that AM fungi significantly bolster plant defense mechanisms against temperature stress through a holistic strategy, encompasses enhanced nutrient uptake, elevated antioxidant activities, and advanced physiological adaptations. Cumulatively, these improvements foster greater plant growth and strengthen tolerance to extreme temperature stresses among a wide array of plant species.

## 5. Effects of AM Fungi on Heavy Metal Stress

AM fungi enhance plant tolerance to heavy metal stress, which can significantly affect root structure and nutrient absorption [171]. The extraradical mycelium of AM fungi extends beyond the nutrient depletion zone of plant roots, thereby expanding the nutrient source and increasing root surface area. This mycorrhizal network significantly enhances the host plant’s resistance to stress and improves tolerance in heavy metal-contaminated soils [172,173]. For instance, Gao et al. (2020) [174] found that the AM fungi *Heterorhizium* sp. enhanced the expression of genes related to specific phosphate transport proteins in *Gossypium hirsutum*, increasing phosphorus uptake by 43.27%. This demonstrates the positive effects of AM fungi on plant nutrient and water absorption, which in turn dilute the lead (Pb) concentrations under stress and improve the host’s tolerance to Pb [175]. 

Table 4 presents the responses of various host plants to specific heavy metal stresses following inoculation with AM fungi. For instance, after 12 months of inoculation with the AM fungus *Mosidustus tubigatus*, the growth of smallflowered needlegrass under lead stress was significantly enhanced, accompanied by a notable increase in lead accumulation within the root system [176]. Liang et al. (2023) [177] observed that AM symbiosis improved the growth characteristics of paper mulberry (*Broussonetia papyrifera*) under moderate Cd stress and enhanced PSI and PSII reactions; it increased ROS levels as a signaling response and maintained ROS balance by boosting root catalase (CAT), peroxidase (POD), and superoxide dismutase (SOD) activities. Under high Cd stress, AM symbiosis promoted the AsA-GSH cycle, reducing cellular damage caused by excessive ROS. Enhanced photosynthesis from this interaction provides more energy and organic matter, aiding the plants in combating oxidative stress and physiological damage from Pb stress [178]. Compared to non-inoculated acacia seedlings, those inoculated with AM fungi showed reduced damage from high Pb concentrations, maintaining higher electron transport rates and PSII photochemical efficiency under Pb stress [179].

AM fungi reduce metal toxicity to plants by mediating interactions with toxic metals in the soil [180,181]. Mycorrhizae act as barriers to metal transport, decrease translocation, and increase the root/shoot Cd ratio [182]. The adsorption of metals onto mycelial walls, attributed to chitin’s strong metal-binding properties [183,184,185], and the metal chelating effects of the glycoprotein glomalin produced by AM fungi can reduce plant metal uptake [186,187,188,189]. However, the impact of AM fungi on metal uptake varies, with some studies showing an increase in metal uptake, while others indicate a decrease [190,191,192,193,194,195,196].

AM fungi can sequester heavy metals within their structures, limiting their translocation to plant cells. They immobilize heavy metals through their mycelia, produce secondary metabolites such as glycoproteins and organic acids, and alter metal bioavailability, thereby affecting both the host plant and its rhizosphere microenvironment [197]. The soil protein glomalin, secreted by AM fungi, has a robust capacity for binding heavy metals and stabilizing them within the soil [198]. AM fungi also directly adsorb heavy metals through their structures, enhancing plant metal absorption [199]. Feng et al. (2023) [200] found that a mixed inoculum of AM fungi from the genera *Rhizophagus*, *Claroideoglomus*, and *Glomus* significantly increased Pb absorption in *Paspalum notatum*, with the Pb content in the roots being 11–197 times higher than in the leaves.

The mechanisms by which AM fungi enhance plant adaptability to soil heavy metal pollution include direct and indirect effects. Direct effects involve structural anchoring, barrier functions, and the expression of AM fungal genes and proteins related to metal transport or detoxification [201,202,203,204]. Indirect effects include altering the rhizosphere microenvironment, changing the morphological structure of the root system, improving the nutrient status, increasing antioxidant enzyme activity, and upregulating the expression of host resistance-related genes and proteins [205,206,207,208,209].

To manage heavy metal stress, plants regulate genes related to absorption and transport, such as ABC transporters (e.g., *AtABCC1 and AtABCC2*), heavy metal ATPases (*HMA*), zinc–iron transport proteins (*ZIP*), and chelation-related genes such as phytochelatin synthase (PC), metallothionein (*MT*), and natural resistance-associated macrophage proteins (e.g., *AtNRAMP6*) [210,211]. Additionally, AM fungi promote plant resistance to heavy metal stress by enhancing photosynthesis, improving nutritional status, and regulating the antioxidant enzyme system. The fungal genes *RintABC1*, *GrosMT1*, and *RintMT1* are involved in heavy metal transport or detoxification [203,204,212], highlighting the significant role of AM fungi in enhancing plant tolerance under heavy metal stress.

**Table 4:** Role of AM fungi in enhancing plant tolerance to heavy metal stress.

Host Plants	AM Fungi Strains	Heavy Metal Stress Type	Responses Related to AM Fungi Inoculation	References
*Bidens parviflora*	*Funneliformis mosseae*	Lead (Pb)	Enhanced oxidative stress defense via increased activity of superoxide dismutase, catalase, ascorbate peroxidase, and glutathione reductase; improved chlorophyll concentration and photosynthesis efficiency; increased root Pb accumulation to protect aerial parts.	[176]
*Broussonetia papyrifera*	*Rhizophagus irregularis*	Cadmium (Cd)	Improved growth and photosynthesis; regulated ROS under low and medium Cd stress; enhanced AsA-GSH cycle under high Cd stress; modulated Cd chelation, soil pH, GRSP content, and phosphorus-related Cd dynamics; differential gene regulation for heavy metal transport.	[177]
Maize (*Zea mays* L.)	*Glomus mosseae*, *Indigenous* P2 *fungal culture*	Cadmium (Cd), Zinc (Zn), Copper (Cu), Lead (Pb), Manganese (Mn)	Experiment 1: Enhanced biomass, reduced Cd, Cu, Zn, and Mn concentrations, indicating protection against metal toxicity. Experiment 2: Varied responses; increased Cu in shoots and Zn in both treatments, increased Pb concentration in roots, no significant change in Cd. Root–shoot translocation of Cu and Zn increased.	[194]
Red Clover	*Glomus mosseae*	Zinc (Zn)	Enhanced Zn uptake at lower levels; reduced translocation to shoots at higher levels; increased P nutrition; hyphae directly absorbed and transferred Zn to roots.	[195]
*Pteris vittata*	*Glomus mosseae*, *Gigaspora margarita*	Arsenic (As)	Phytoremediation techniques are receiving more attention as decontaminating strategies. Increased As concentration in pinnae, higher P concentration, enhanced As translocation and plant growth.	[196]
Kenaf (*Hibiscus cannabinus* L.)	*Rhizophagus aggreratus*	Cadmium (Cd)	Improved nutrient transport efficiency and plant growth; increased cell wall polysaccharide content binding Cd in roots, reducing its transport to aerial parts; enhanced soil balcomycin content aiding in Cd chelation; upregulated expression of genes like *Hc.GH3.1*, *Hc.ARK*, and *Hc.PHR1* enhancing Cd tolerance.	[213]
Rice (*Oryza sativa*)	*Funneliformis mosseae* (Fm), *Rhizophagus intraradices* (Ri)	Cadmium (Cd)	Decreased root and shoot Cd concentrations, especially with Ri. Altered expression of Cd transporters (*Nramp5*, *HMA3*) influencing Cd uptake. Ri treatment led to higher abundance of Actinobacteria, reducing soil Cd availability.	[214]
Sunflower (*Helianthus annuus* L.)	*Funneliformis mosseae*, *Rhizophagus intraradices*, *Claroideoglomus etunicatum*	Cadmium (Cd)	Increased growth, chlorophyll content, and cell membrane stability. Enhanced antioxidant enzyme activities, increased proline and total phenols, reduced lipid peroxidation and hydrogen peroxide production. Mitigated negative impacts on fatty acids and phosphatase activities under cadmium stress.	[215]
*Medicago sativa*	*Glomus aggregatum*, *G. intraradices*, *G. elunicatum*, *G. versiforme*	Cadmium (Cd)	Increased shoot and root biomass, especially in combination with biochar. Enhanced N, P, K, Ca uptake; reduced Cd concentration in plant tissues.	[216]

## 6. Effects of AM Fungi on Waterlogging Stress

Waterlogging constitutes a significant abiotic stress that profoundly influences plant growth, productivity, and geographical distribution, as established by seminal research [217,218,219]. This stressor induces anoxia and hypoxia within the root zone, resulting in reduced hydraulic conductivity and stoma constriction, which in turn severely impair internal water management, photosynthetic activity, and nutrient assimilation, thereby diminishing plant vitality [219,220,221]. Plants respond to these challenges by deploying morphological, anatomical, and physiological adaptations, such as the development of adventitious roots and lenticels, to enhance respiration and optimize oxygen levels within the roots [219,221,222].

AM fungi, as obligate symbionts, pervade aquatic and wetland ecosystems and form symbiotic relationships with 70–90% of terrestrial plants [223,224]. These fungi exhibit notable resilience in environments subjected to continuous or seasonal waterlogging, including ecosystems such as mangroves, salt marshes, riverbanks, floodplains, and peat swamp forests [225,226,227,228,229,230,231]. Current research underscores that AM fungi significantly improve the adaptability of crops to waterlogged conditions by enhancing nutrient absorption, promoting growth and biomass, and accelerating the succession of aquatic plant communities. Table 5 presents a comprehensive overview of the responses of various host plants to different flooding stresses following inoculation with AM fungi. For example, in an Indonesian tropical peat swamp forest, *Dyera polyphylla* plants inoculated with *Glomus clarum* and *Gigaspora decipiens* demonstrated enhanced tolerance to waterlogging and elevated nitrogen and phosphorus levels [232]. AM fungi also facilitate phosphorus uptake in diverse plant species including *Panicum hemitomon* and *Oryza sativa* [231,233,234,235,236,237,238]. Furthermore, AM fungi inoculation boosts the accumulation of organic osmolytes such as sugars and proline, mitigating the production of toxic byproducts like ethanol from anaerobic respiration [236,239,240,241]. Inoculation with *Diversispora spurca* enhances the activity of antioxidant enzymes (SOD and CAT) in citrus species, reducing oxidative stress [242,243]. Additionally, inoculation with *F. mosseae* increases chlorophyll content and photosynthetic efficiency in peach leaves [240].

AM fungi inoculation is associated with improved photosynthetic capacity and water uptake, benefiting stomatal conductance under waterlogged conditions [235,244]. This effect is particularly evident in tomato plants, where AM fungi symbiosis increases root hydraulic conductivity, linked to the upregulation of both plant and fungal aquaporins, SIPIP1;1 and GintAQP1, respectively [245]. However, the impact of AM fungi is not universally beneficial; while it can promote community establishment along riverbanks and improve adaptability in salt marshes, some studies report potential growth inhibition in certain plant species under specific conditions [231,246,247,248,249].

**Table 5:** Role of AM fungi in enhancing plant tolerance to waterlogging stress.

Host Plants	AM Fungi Strains	Waterlogging Stress Condition	Responses Related to AM Fungi Inoculation	References
*Dyera polyphylla*	*Glomus clarum*, *Gigaspora decipiens*	Permanent and seasonal waterlogging	Enhanced tolerance to waterlogging stress; increased nitrogen and phosphorus content	[232]
*Pterocarpus officinalis*	Glomus intraradices	Permanent and seasonal waterlogging	Increased phosphorus uptake	[250]
*Panicum hemitomon Schultes* and *Leersia hexandra Schwartz*	*Acaulospora trappei*, *Scutellospora heterogama*, *A. laevis*, *Glomus leptotichum*, *Glomus etunicatum and Glomus gerdemannii.*	following rooting-zone flood regimes	Increased phosphorus uptake	[233]
*Panicum hemitomon Schult* L. and *Typha latifolia* L.	AM fungal assemblages—collected from different vegetation communities in a Florida wetland	flooded conditions	Improved some plant-growth and P-nutrition parameters at lower P levels relative to nonmycorrhizal controls, but generally conferred no benefit or was detrimental at higher P levels.	[234]
*Typha latifolia*	Not Specified(using fieldcollected soils were maintained for 12 weeks to increase the biomass of mycorrhizal fungi.)	Inundated soils at three levels of phosphorous availability conditions for 11 weeks.	Increased phosphorus and nitrogen uptake	[235]
*Prunus persica Batsch*	Not Specified	3 days of flooding	Increased phosphorus, nitrogen uptake and root activity; inhibited ethanol	[236]
*Aster tripolium*	*Glomus geosporum*	tidal flooding conditions for 56 d	Improved osmotic regulation through accumulation of sugars and proline; enhanced oxidative stress defense	[239]
*Poncirus trifoliata*	*Diversispora spurca*	waterlogging	Increased superoxide dismutase and catalase activities in leaf and root under both NWL and WL, thereby, resulting in lower oxidative damage in terms of malondialdehide concentration.	[242]
*Citrus junos*	*Diversispora spuraca*	Waterlogging for 37 d	Significantly increased root catalase (CAT) activity in non-stressed seedlings and increased root soluble protein concentration and leaf CAT activity in waterlogged seedlings, thereby inducing lower oxidative damage.	[243]
*Prunes persica* (L.) *Batsch*	*Funneliformis mosseae*	Waterlogging for 12 d	Increased accumulation of proline; increase in P5CS activity and a decrease in δ-OAT and ProDH activity; enhanced chlorophyll concentration and photosynthesis efficiency	[240]

## 7. Effects of AM Fungi on Plant Resistance to Biotic Stresses

Plant pathogenic fungi frequently inflict significant damage on plant growth. For instance, *Botrytis cinerea* infection in lettuce leads to reduced levels of chlorophyll, carotenoids, and carbohydrates [251]. Similarly, *Fusarium graminearum* infection in wheat is associated with increased concentrations of phenolic compounds and amino acids [252]. In contrast, AM fungi have been documented to exert beneficial control over diseases caused by pathogenic fungi, bacteria, viruses, and nematodes [253]. AM fungi significantly bolster host plant resistance against various biotic stresses and mitigate damage from pathogen infections. Research has demonstrated that AM fungi inoculation considerably enhances the activity of antioxidant enzymes and the content of antioxidants in plants, such as ascorbate peroxidase (APX), monodehydroascorbate reductase (MDHAR), superoxide dismutase (SOD), ascorbic acid (ABA), and glutathione (GSH). These enhancements help plants resist oxidative damage induced by pathogens and reduce disease symptoms [254].

Table 6 delineates the responses of various host plants to a range of biotic stresses following inoculation with AM fungi. For instance, Villani et al. reported that inoculating *Cynara cardunculus* with *Glomus vismiae* significantly increased the activity of defensive enzymes like APX, MDHAR, and SOD, and elevated levels of ASA and GSH, which led to decreased H2O2 concentrations and reduced lipid peroxidation, thus strengthening the plant’s resistance to fungal pathogens [255]. Lin et al. investigated the impact of AM fungi inoculation on banana seedlings and the expression of defense-related genes. Their findings highlighted that inoculation not only spurred plant growth but also triggered the expression of disease resistance genes, offering effective protection against Fusarium wilt [256]. Similarly, Hygesen’s research indicated that inoculation with Glomus species not only promoted plant growth but also heightened resistance to root rot, with *G. cerebrosum* showing greater effectiveness than *G. clarum* [257,258]. Campo et al. assessed the response of 12 rice varieties to AM fungi inoculation, noting that all varieties established a symbiotic relationship with AM fungi. The growth-promotion effects varied based on the AM fungi traits, and this interaction also influenced resistance to rice blast differently, increasing infection rates in some susceptible varieties [259]. Nanjundappa et al. explored the synergistic effects of AM fungi and plant growth-promoting rhizobacteria (e.g., *Bacillus* spp.), which are vital for enhancing soil fertility and plant health. This dual inoculation with AM fungi and Bacillus subtilis notably increases nutrient uptake and offers improved protection against pathogens and resistance to salinity and heavy metal toxicity. Compared to single inoculations, this combination could reduce the usage of nitrogen, phosphorus, and potassium (NPK) fertilizers by up to 50% without compromising plant growth or yield, suggesting a significant avenue for future research [260].

AM fungi demonstrate significant preventive effects against a variety of pests, including root-knot nematodes and pea aphids, primarily through enhanced plant nutritional status and activation of stress resistance genes. Studies have shown that AM fungi inoculation boosts plant growth, increases nutrient absorption, and modulates hormonal signaling and defense enzyme activities, such as peroxidase (POD) and superoxide dismutase (SOD). These biological enhancements lead to considerable increases in biomass and nutrient content, specifically nitrogen and phosphorus, thereby strengthening the plants’ defense mechanisms against biotic stressors [261,262,263]. For example, research by Jin Zhi-Bo et al. revealed that AM fungi inoculation significantly reduced damage from southern root-knot nematodes in tomatoes. This finding is supported by field studies indicating that tomato seedlings inoculated with a mixture of AM fungi strains showed a decrease in nematode infestation, with reductions in galls and egg masses of 22.8% and 23.5%, respectively [263,264,265]. Additionally, coffee plants treated with various AM fungi strains, including Huall-pache, Do-cat, and Mo-cat, exhibited notable decreases in nematode infection severity, with average reductions ranging from 38.3% to 52.5% [266]. Further investigations also demonstrate that AM symbiosis can confer tolerance to Nacobbus aberrans, a pathogen that adversely affects the growth and yield of many horticultural crops. Controlled experiments with three AM fungi strains—*Rhizophagus intraradices* B1, *Rhizophagus intraradices* A2, and *Funneliformis mosseae*—were effective in reducing N. aberrans populations in pepper plants. The results included enhanced mycorrhization, significant reductions in nematode populations, and decreased physiological stress markers such as proline, phenolic compounds, and sugars, all of which collectively improve plant health and productivity [267].

These results highlight the crucial role of AM fungi in enhancing plant health and providing protection against a diverse range of pests and pathogens, including root-knot nematodes, pea aphids, various fungi, and bacterial pathogens. This comprehensive defense, facilitated by multiple mechanisms, underscores the significant potential of AM fungi in advancing sustainable agricultural practices. AM fungi contribute to increased plant resilience against diseases and pests, alleviate the impacts of biotic stresses, and promote root colonization by beneficial microorganisms. Furthermore, AM fungi reduce plant vulnerability to soil-borne pathogens and activate mechanisms of resistance or tolerance against a broad spectrum of bacterial challenges. These effects are mediated through the upregulation and downregulation of specific genes, eliciting both localized and systemic plant responses [196,268,269,270].

**Table 6:** Role of AM fungi in enhancing plant tolerance to biological stress.

Host Plants	AM Fungi Strains	Biological Stress Type	Responses Related to AM Fungi Inoculation	References
Artichoke (*Cynara scolymus* L.)	*Glomus vicosum*	Verticillium wilt caused by *Vertcillium dahliae*	Increased activity of antioxidant enzymes: ascorbate peroxidase (APX), monodehydroascorbate reductase (MDHAR), and superoxide dismutase (SOD)	[255]
Banana (*Musa acuminata* ‘Cavendish’ cv. ‘Brail’)	*Rhizophagus irregularis* (Ri)	Fusarium wilt caused by *Fusarium oysporum* f. sp. *cubense*	Increased plant dry weights in stem, leaf, and overall; up-regulation of defense-related genes (*POD*, *PAL*, *PYR*, *HBP-1b*); enhanced expression of growth-related genes (*IAA*, *GH3*, *SAUR*, *ARR8*).	[256]
Pea (*Pisum sativum*)	*Glomus intraradices*, *Glomus claroideum*	Pea root-rot caused by *Aphanomyces euteiches*	Reduced disease incidence, especially with *G. intraradices*; enhanced mycorrhizal development and potential induction of tolerance against pea root-rot.	[257]
Rice (*Oryza sativa*, japonica subspecies)	*Funneliformis mosseae*, *Rhizophagus irregularis*	Blast disease caused by *Magnaporthe oryzae*	Enhanced root colonization, especially by *R. irregularis*; increased Pi content in leaves; improved growth, productivity, and blast resistance, varying by rice cultivar; significant increase in grain yield in field conditions	[259]
Eggplant	*Glomus mosseae* (Gm), *Ggaspora gigantea* (Gg)	Root-knot nematode (*M. javanica*)	Reduced root-knot nematode infestation; improved growth traits and fruit biochemical content; higher levels of mycorrhization (68.20%); outperformed single treatments in most traits	[261]
*Medicago truncatula* (‘Jemalong’, line A17)	*Rhizophagus irregularis*	Pea aphid (*Acyrthosiphon pisum*)	Increased preference by adult aphids for highly AM fungi-colonized plants; mixed age aphids showed reduced weight on low AM colonized plants, indicating possible priming by AM fungi; gene expression changes in roots related to gibberellin metabolism.	[271]
*Ageratina adenophora*	*Claroideoglomus etunicatum*, *Septoglomus constrictum Claroideoglomus etunicatum*, *Septoglomus constrictum*	*Aphis gossypii*	Enhanced growth (increased aboveground and root biomass) and resistance to *A. gossypii*, elevated polyphenol oxidase, jasmonic acid, and flavonoid levels, and reduced *A. gossypii nymph* survival and density, with *C. etunicatum* showing a greater effect than *S. constrictum*.	[272]
*Plantago major* and *Poa annua*	*Rhizoglomus irregulare*	Generalist aphid (*Myzus persicae*)	Slight increases in sucrose proportions and shifts in amino acid profiles in phloem exudates. Negative effects on aphid survival in *P. major*, but positive effects in *P. annua* on the next aphid generation.	[273]

## 8. Effects of AM Fungi on Crop Yield

Recent studies have demonstrated that AM fungi substantially boost crop yield and biomass under various environmental stresses by enhancing nutrient uptake and physiological processes in plants [231]. Lehmann and Rillig observed that AM fungi markedly improve the nutritional status of plants, which in turn augments their growth and productivity [274]. Furthermore, soil physicochemical properties, such as texture and pH, critically influence AM fungi functionality, thereby determining nutrient availability and solubility [231,275]. Under saline conditions, *Glomus etunicatum outperforms G. mosseae* and *G. intraradices*, underscoring the significance of selecting appropriate AM fungi species and understanding their interactions with host plants [276]. 

Table 7 illustrates the role of AM fungi inoculation in enhancing the yields of various crops.Studies have shown that AM fungi enhance root colonization and increase phosphorus and nitrogen absorption, significantly boosting yields in soybeans and cotton when compared to conventional chemical fertilization [277]. Additionally, different AM fungi species have been shown to significantly improve growth and nutrient uptake in various wheat cultivars under salt stress, particularly in high-salinity environments [278]. In arsenic-contaminated soils, AM fungi have improved arsenic tolerance, phosphorus uptake, and crop yields in rice [279].Further research indicates that AM fungi enhance crop resilience against diverse environmental stresses. For instance, AM fungi have been found to improve water and nutrient absorption in mung beans under drought conditions [280] and to increase nitrogen, phosphorus, and total chlorophyll contents in flax under salt stress, which supports enhanced crop yield and physiological properties [281]. In other studies, AM fungi have been shown to boost yield and nutrient absorption in chickpeas and maize under both rain-fed and irrigated conditions, as well as enhancing the essential oil yield and composition in basil [282,283,284]. Moreover, selecting native AM fungi species has significantly increased cassava’s tolerance to nematode and water stress, highlighting the crucial role of AM fungi in enhancing crop yields and ecological adaptability [285].

These findings underscore the pivotal role of AM fungi in boosting agricultural productivity, improving nutrient absorption efficiency, and augmenting crop adaptability to various abiotic stresses. Utilizing AM fungi allows for more effective resource management in modern agriculture, enhancing the environmental adaptability and productivity of crops. This not only demonstrates the substantial potential of AM fungi in agricultural applications but also highlights their importance in global sustainable agricultural development and climate change mitigation.

## 9. Application Potential of AM Fungi

AM fungi offer considerable benefits in terms of soil improvement and crop yield and quality enhancement. AM fungi establish a symbiotic relationship with crop roots, effectively boosting the nutrient absorption capacity and sustaining high yields even in soils of low fertility [286,287]. Additionally, AM fungi enhance soil health by improving soil structure and increasing the diversity of beneficial microorganisms, which in turn augments the nutritional quality and growth performance of crops [288]. AM fungi elevate the availability of nitrogen, phosphorus, and potassium in the soil and enhance microbial diversity, thus bettering the growth environment for crops.

For instance, in maize, AM fungi application notably enhanced root growth, yield, and grain quality, particularly in sandy and saline soils, by increasing soil nutrient content and microbial diversity, demonstrating AM fungi’s potential in sustainable agricultural practices [289]. In wheat, AM fungi inoculation improved phosphorus absorption and utilization efficiency, especially under arid conditions, maintaining productivity by enhancing water use efficiency [290]. Lettuce grown in greenhouses with AM fungi inoculation exhibited significant growth rate increases and nutritional quality improvements, particularly in the uptake of minerals such as zinc and phosphorus [291]. Similarly, the use of AM fungi in citrus crops, specifically *R. irregularis* and *F. mosseae*, has been shown to significantly boost the growth and health of lime seedlings, thus enhancing crop yield and quality [286].

While the majority of studies highlight the symbiotic benefits of arbuscular mycorrhizal (AM) fungi to host plants, demonstrating enhanced growth and stress tolerance, some research underscores the complexities of this relationship, particularly in terms of carbon dynamics. Colonization by AM fungi significantly alters carbon partitioning and metabolism within the host plant [292]. In plants colonized by these fungi, there is typically an increase in the rate of carbon assimilation, the exportation of photosynthates from leaves, and the absorptive capacities of roots compared to non-colonized counterparts. However, these benefits come at a cost. The obligate symbiotic fungi utilize hexose sugars from their hosts for growth, maintenance, and reproduction, which can impose a substantial carbon cost on the host, especially under conditions where the fungi provide minimal nutritional benefit [292]. For instance, leaves of non-mycorrhizal plants have been found to contain higher starch reserves compared to those of mycorrhizal plants [293], and in mycorrhizal *Panicum coloratum*, the concentration of photosynthates in stem tissues is double that found in non-mycorrhizal plants [294].

Moreover, AM fungi are particularly valuable in water-saving agriculture and organic farming systems [286,291,295], helping reduce environmental pollution by diminishing the reliance on chemical fertilizers and enhancing crop stress resistance and adaptability [286,295]. Hence, AM fungi are not merely crucial biological agents for boosting crop yield and quality but also pivotal technologies for promoting sustainable agricultural development.

## 10. Conclusions

**Figure 4:**
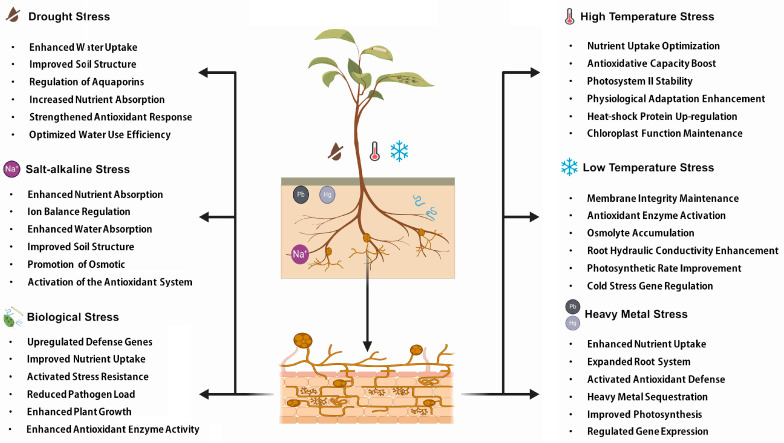
Mechanisms of action of AM fungi in enhancing plant resistance to biotic and abiotic stresses. (All components, with the exception of the text, were created using BioRender.).

This review encapsulates the pivotal findings on the role of AM fungi in enhancing crop stress tolerance. As shown in Figure 4, AM fungi significantly bolster plant resilience to adversities such as salinity, drought, heavy metal contamination, extreme temperatures, and pathogen attacks. By promoting root growth, improving nutrient uptake, and modulating antioxidant responses, AM fungi enhance the overall stress resistance of plants.

Future prospects for the application and development of AM fungi in agriculture focus on enhancing the benefits and application scope of AM fungi. With technological advancements, the development and application optimization of new AM fungi formulations will become research focal points. Future studies should explore the mechanisms through which AM fungi regulate plants under various stress conditions and investigate the synergistic effects of AM fungi with other agricultural management practices like fertilization and irrigation to maximize their contribution to crop production.

## Figures and Tables

**Table 1:** Role of AM fungi in enhancing plant drought tolerance.

Host Plants	AM Fungi Strains	Drought Stress Condition	Responses Related to AM Fungi Inoculation	References
*Triticum aestivum*;	*Funneliformis mosseae*, *F. geosporum*	Natural drought condition, with relative soil water content at 22% of control.	Increased relative water content (RWC) in leaves and soil, mitigated structural and functional damage to PSII and PSI under drought stress, and enhanced photochemical efficiency.	[83]
*Zenia insignis*	*Funneliformis mosseae*, *Rhizoglomus intraradices*, *sispora versiformis*	Drought stress treatment involved irrigation every 7–10 days to maintain soil moisture at 35–45% of field capacity over 4 months.	Enhanced plant biomass and antioxidant capacity, plant biomass, P uptake, and osmolytes (like soluble sugars and proline).	[84]
*Ephedra foliata Boiss*	*Claroideoglomus etunicatum*, *Rhizophagus intraradices*, *Funneliformis mosseae*	Drought stress induced by regulating Jensen’s nutrient solution supply, withholding water.	Increased plant biomass and antioxidant capacity, enhanced N metabolism, P uptake, osmolytes (like proline and glucose), improved nutrient absorption (K, Mg, Ca), higher phytohormone levels (IAA, IBA, GA, ABA), and enhanced P metabolism.	[85]
*Zea mays* L.	*Glomus versiforme*	Natural drought condition.	Enhanced plant biomass and antioxidant capacity, improved chlorophyll and carotenoid content, increased mineral uptake and assimilation, up-regulation of the antioxidant system, and elevated levels of compatible solutes (like proline, sugars, and free amino acids) under moderate and severe drought conditions.	[86]
Soybean (*Glycine max* L.)	*Septoglomus constrictum*, *Glomus* sp. and *Glomus aggregatum*	Soil allowed to dry to 7% volumetric moisture over 7 days, with daily water resupply; mycorrhizal plants harvested 60 days post-planting.	Enhanced plant biomass and antioxidant capacity, improved water content and nutrient concentrations (P and N), and maintained levels of osmotic metabolites (like soluble sugars and proline) under drought stress.	[87]
*Catalpa bungei* C.A.Mey.	*Rhizophagus intraradices*	moderate drought (50%), and severe drought (30%) of field capacity.	Enhanced plant growth and antioxidant capacity, increased photosynthetic efficiency and nutrient absorption (N, P), improved soil structure (GRSP, macro-aggregates). Reduced root/shoot ratio and modulated hormone levels (higher IAA, GA_3_; lower ABA, ZT), alleviating oxidative stress under drought conditions.	[88]
*Spinacia oleracea* L.	Commercial inoculum (Clonex^®^ Root Maximizer)	Drought stress (DS) maintained at 30% of field capacity; no drought stress (NDS) at normal field capacity levels.	Enhanced growth parameters (shoot and root weight, length), increased photosynthetic activity (higher chlorophyll content, photosynthetic rate, stomatal conductance), and improved nutrient content (N, P, K).	[89]
*Oryza sativa* L.	*Funneliformis mosseae*, *F. geosporus*, *Claroideoglomus claroideum*, *Glomus microaggregatum*, *Rhizophagus irregularis*	Initial 42 days well-watered; drought initiated at 42 days with cycles of drying and rewetting, reaching soil water potential down to −80 kPa; recovery phase post −90 DAP.	Improved nutrient uptake (especially P), increased stomatal conductance and chlorophyll fluorescence, and modulated hormone levels (higher IAA, varied ABA) under drought stress. Reduced grain yield loss and maintained shoot and root biomass.	[90]

Note: IAA, indole-3-acetic acid; GA_3_, gibberellins 3; ABA, abscisic acid; ZT, zeatin.

**Table 2:** Role of AM fungi in enhancing plant tolerance to salt stress.

Host Plants	AM Fungi Strains	Responses Related to AM Fungi Inoculation	References
*Casuarina obesa* (Miq.)	*Rhizophagus fasciculatus*, *Rhizophagus aggregatum*	Enhanced survival rate, plant height, and biomass; increased chlorophyll and proline accumulation.	[133]
*Trigonella foenum-graecum* L.	*Glomus intraradices*	Reduced cellular and ultrastructural damage under salt stress, lower lipid peroxidation and electrolyte leakage, increased osmolytes (glycinebetaine, sugars), polyamines, and α-tocopherol enhancing ionic balance and stress tolerance.	[134]
Tomato (*Lycopersicon esculentum Mill.*) ‘Pello’ (salt-tolerant) and ‘Marriha’ (salt-sensitive)	*Glomus mosseae*	Enhanced fruit yield, shoot dry matter, and mineral content (P, K, Zn, Cu, Fe), while reducing Na^+^ concentration in tomatoes, leading to greater salt stress tolerance and higher root colonization.	[135]
Tomato (*Solanum lycopersicum* L.)	*Glomus* sp. mixture	Enhanced nutrient uptake and root system dry matter, maintaining higher growth rates under moderate-to-severe salt stress compared to non-inoculated plants.	[136]
*Pisum sativum* L.	*Funneliformis mosseae* and *R. intraradices*	Enhanced nutrient uptake, osmolyte accumulation, and reduced electrolyte leakage, leading to improved biomass production, chlorophyll synthesis, yield, and growth in pea under salinity stress. The consortium of *R. fasciculatum* and *Gigaspora* sp. was particularly effective.	[137]
*Stevia rebaudiana Bertoni*	*Rhizophagus intraradices*, *consortium*	Notable improvements in growth, physiological responses, and antioxidant enzyme activities in *Stevia rebaudiana*, reducing the negative impacts of salt stress. The AM fungi consortium demonstrated greater efficacy than *Rhizophagus irregularis* in enhancing plant resilience to salinity.	[138]
*Cucumis sativus* L.	*Claroideoglomus etunicatum*, *Rhizophagus intraradices*, *Funneliformis mosseae*	Mitigated salt stress in cucumbers by enhancing biomass, pigment synthesis, and antioxidant enzyme activities; increased ascorbic acid content and accumulation of phenols and proline helped neutralize superoxide radicals, while increased levels of jasmonic acid, salicylic acid, and essential minerals were observed alongside a reduced uptake of Na^+^.	[139]
Cotton (Xinluzao 45)	*Funneliformis mosseae*, *Rhizophagus irregularis*, *Claroideoglomus etunicatum*	Improved photosynthesis, increased CO_2_ concentration, transpiration, and energy use efficiency, significantly enhancing cotton growth, plant height, and root length under saline–alkali stress. *Funneliformis mosseae* showed the most significant improvement in growth and photosynthetic activity.	[140]
*Leymus chinensis*	*Funneliformis mosseae*, *Rhizophagus intraradices*, *Diversispora versiformis*, *Acaulospora scrobiculata*	Improved stress tolerance by enhancing growth, nutrient absorption, ion balance, and photosynthesis, particularly with *Funneliformis mosseae*, *Rhizophagus intraradices*, and mixtures showing greater benefits under high stress.	[141]
*Triticum aestivum* L.	*Acaulospora laevis*, *Funneliformis geosporum*, *Funneliformis mosseae*, *Cetraspora armeniaca*	Mitigated alkalinity stress in wheat by improving germination, biomass, photosynthetic pigments, and nutrient uptake (K, N, P). It also reduced lipid peroxidation and enhanced the activity of stress-related enzymes like catalase and peroxidase, contributing to better overall productivity and crop yield.	[142]
Processing Tomato (*Lycopersicon esculentum* Mill.)	*Mixed fungi including Glomus clarum* and *Glomus intraradices*	Improved growth, enhanced nutrient absorption (increased N uptake and reduced Na^+^ uptake), and optimized physiological processes under saline–alkali stress. This led to increased concentrations of soluble solids, vitamin C, soluble sugars, and lycopene in fruits, improved ion ratios (K^+^/Na^+^, Ca^2+^/Na^+^, Mg^2+^/Na^+^) in leaves and stems, and protected photosynthetic organs. AM fungi also boosted the chlorophyll content, photosynthetic rate, stomatal conductance, and transpiration rate, while optimizing the microbial community in the rhizosphere.	[143]
*Leymus chinensis*	BGC HEB02	Mitigated growth inhibition under combined alkali and drought stresses by enhancing osmotic adjustment, improving ionic balance, and counteracting ion toxicity and oxidative damage.	[144]
*Lolium arundinaceum*	*Funneliformis mosseae*, *Claroideoglomus etunicatum*	The interaction between *Epichloë endophytes* and AM fungi significantly enhanced tall fescue’s resistance to saline–alkali stress by increasing biomass, nutrient uptake (organic carbon, total N, P), and K^+^ accumulation, while reducing Na^+^ concentrations.	[145]
*Lycium ruthenicum*	*Funneliformis mosseae*, *Rhizophagus intraradices*	Significantly enhanced growth and saline–alkaline resilience in black wolfberry, improving chlorophyll b and P absorption, reducing reactive oxygen species, and increasing abscisic acid accumulation, aiding in better ion management and stress response.	[146]
*Puccinellia tenuiflora*	*Rhizophagus intraradices*	Increased biomass and altered metabolic responses under alkali stress, enhancing levels of amino acids, organic acids, flavonoids, sterols, and plant hormones (salicylic acid, abscisic acid), which improved osmotic adjustment, cell membrane stability, and stress resilience.	[147]

**Table 3:** Role of AM fungi in enhancing plant tolerance to extreme temperature stress.

Host Plants	AM Fungi Strains	Extreme Temperature Stress Type	Responses Related to AM Fungi Inoculation	References
Cucumber (*Cucumis sativus* L. cv. Zhongnong No. 26)	*Rhizophagus irregularis* (isolate PH5)	Exposure to cold-stress conditions at 15/10 °C (day/night) for a period of 14 days	Countered the negative effects of cold stress by enhancing chlorophyll content, net photosynthetic rate, and photochemical quenching. Reduced non-photochemical quenching and moderated the increase in sugar contents, indicating improved photosynthetic efficiency and carbon sink strength.	[152]
Watermelon (*Citrullus lanatus*) cv. “Crimson Sweet” and “Charleston Gray”	*Glomus intraradices*	Subjected to chilling treatment in chambers maintained at 4 ± 0.5 °C for durations of 12 and 36 h	Significantly enhanced root and shoot dry mass, improved chlorophyll content and photosynthesis efficiency, and reduced oxidative stress markers such as H_2_O_2_ and MDA. Decreased electrolyte leakage and increased peroxidase activity, enhancing chilling resistance.	[161]
*Kobresia filicina*, *K. myosuroides*, *Polygonum viviparum*, *Alnus nitida*, *Betula pendula*, *Pinus sylvestris*, *Trifolium repens*	*Cenococcum geophilum*	Exposed to extreme cold conditions at +5 °C, −10 °C, −20 °C, −40 °C, and −50 °C, down to −125 °C.	Improved cold stress tolerance by enhancing root and shoot biomass, chlorophyll content, and photosynthetic efficiency. Significantly decreased oxidative stress markers like H_2_O_2_ and MDA, and moderated electrolyte leakage. Demonstrated exceptional resilience in *K. myosuroides*.	[162]
Snapdragon (*Antirrhinum majus* L.) ‘Red and White’	*Funneliformis mosseae* and *Glomus versiforme*	Cold-stress conditions of 14/4 °C (day/night) sustained for 7 days.	Enhanced resistance to low-temperature and weak-light stress through physiological and transcriptomic responses.	[163]
*Impatiens walleriana* ‘Super Elf (Rose red)’	*Funneliformis mosseae*, *Glomus versiforme*	Sub-low temperature treatment set at 12 °C/8 °C (day/night).	Improved plant growth and enhanced photosynthetic efficiency under sub-low temperature stress. Increased Fv′/Fm′, Y(II), and qP, while reducing NPQ, ROS (O_2_^−^ and H_2_O_2_) accumulation, and cell membrane lipid peroxidation damage, indicating enhanced cold tolerance.	[164]
Four pearl millet lines	*Rhizophagus aggregatus* and *Funneliformis mosseae*	Heat-stress conditions of 37/32 °C (day/night) over a span of 60 days.	Improved plant growth and physiological responses under temperature stress. Increased chlorophyll concentration, root and shoot dry weight, especially under high temperature conditions, and enhanced soil aggregation. *Funneliformis mosseae* was more effective in promoting root colonization.	[165]
Processing Tomato (Genotypes: ‘Everton’, ‘Pearson’, ‘H3402’)	*Funneliformis mosseae*, Paraburkholderia graminis C4D1M	Chilling treatment executed at 1 °C for 24 h.	Reduced electrolytic leakage and improved efficiency of photosystem II after chilling stress. Enhanced seedling regrowth and photosystem II efficiency in a consortium with *P. graminis*. Specific improvement in modern genotypes under consortium treatment.	[166]
*Zea mays*	*Rhizophagus intraradices*, *Funneliformis mosseae*, and *F. geosporum*	High temperature stress conditions at a stable 44 °C ± 0.2 °C.	Enhanced photosynthetic activity, increased chlorophyll content, and improved overall plant growth under high temperature (44 °C). This included better quantum efficiency of PSII, higher net photosynthesis rate, and greater morphological development (leaf width, plant height, cob number).	[167]
Tomato (*Solanum lycopersicum* L.), Pepper (*Capsicum annuum* L.), Cucumber (*Cucumis sativus* L.)	*Rhizophagus irregularis*, commercial inoculant MYCOGEL (Agrocode Biosciences LTD, Almeria, Spain)	Severe heat stress with temperatures escalating to a peak of 45.6 °C.	Significantly improved the endurance, vigor, productivity, and fruit quality under severe heat stress by applying an ultra-pure in vitro-produced AM fungi concentrate directly to the roots at transplanting, simulating drip irrigation.	[155]
Maize (*Zea mays* L. cv. Navjot)	A mixed culture of AM fungi, primarily consisting of various Funneliformis species, was used	Recorded extreme summer conditions in May 2018, with maximum daily temperatures ranging between 43 and 44 °C in Indore (22°44′ N).	Enhanced PSII heterogeneity by facilitating the conversion of inactive β and γ centers to active α centers, and QB non-reducing centers to reducing centers, improving photosynthetic efficiency and stress resilience under high temperature stress.	[168]
*Cucumis sativus* L.	*Diversispora versiformis*	Heat-stressed environment characterized by 38 °C/30 °C (day/night) for a short-term (80 h) treatment.	Improved growth parameters (plant height, stem diameter, biomass), chlorophyll index, and osmolyte levels (sucrose, fructose, glucose, betaine, proline) under short-term heat stress. Up-regulated PIPs and Hsp70 gene expressions, enhancing heat tolerance.	[169]
Lettuce (*Lactuca sativa* L., cv. Shuangzi)	*Funneliformis mosseae*	High temperature stress condition at 35 °C.	Enhanced resilience to high temperature (35 °C) by improving chloroplast ultrastructure and photosynthetic efficiency. Increased chlorophyll a and b contents, net photosynthetic rate, and transpiration rate. Better maintenance of photosynthetic performance index and fluorescence parameters, suggesting protection against heat-induced PSII damage and improved energy fluxes.	[170]

**Table 7:** Role of AM fungi in enhancing plant yield.

Host Plants	AM Fungi Strains	Growth Condition	Responses Related to AM Fungi Inoculation	References
*Glycine max* L. and *Gossypium hirsutum* L.	*Rhizophagus clarus*	*Field conditions*	AM fungi inoculation increased around 20% of root colonization in both soybean and cotton; increased P and nitrogen content in plants, leading to higher yield.	[277]
Wheat genotypes of Roshan, Kavir and a mutated line of Tabasi	*Glomus etunicatum*, *G. mosseae*, *G. intraradices*	*Nutrient uptake under field saline conditions*	*Glomus etunicatum* > *G. mosseae* > *G. intraradices*. Enhanced wheat dry weight and grain yield; improved phosphorus uptake.	[278]
*Lycopersicon esculentum* L. cv. Zhongzha105	*Glomus mosseae*	*Laboratory simulated salt stress*	AM fungi alleviated salt-induced reduction in root colonization, growth, chlorophyll content, and fruit yield of tomato plants.	[276]
*Oryza sativa* L.	*G. geosporum*, *G. intraradices*	*Arsenic-contaminated soil*	Significant effects on grain As concentration, grain yield, and grain P uptake; enhancement with suitable AM fungi.	[279]
*Pterocarpus officinalis* (Jacq.)	*Glomus intraradices*, *Bradyrhizobium* sp.	*Flooding condition*	Significant increases in yield, root colonization, and shoot phosphorus content.	[250]
*Cicer arietinum* L.	*Glomus intraradices* Shench&Shimith	*Rain-fed conditions*	Enhanced yield, root colonization, and phosphorus content in seed and shoot; effective in combined applications.	[282]
*Ocimum basilicum* L.	AM fungi (PGPR, AM fungi, and PGPR + AM fungi)	*Bolu ecological conditions*	Improved essential oil yield and composition; superior results compared to control in yield parameters.	[283]
*Zea mays* L.	*Glomus intraradices*	*Field conditions*	Improved productivity and growth comparable to conventional treatments; enhanced phosphorus availability.	[284]
*Linum usitatissimum* L.	*Claroideoglomus etunicatum*, *Funneliformis mosseae*, *Glomus aggregatum*	*Irrigation water salinity*	Increased chlorophyll content, nitrogen and phosphorus uptake, seed and stem fiber yield under salt stress conditions.	[281]
